# The value of hepatic resection in metastasic renal cancer in the Era of Tyrosinkinase Inhibitor Therapy

**DOI:** 10.1186/s12893-016-0163-0

**Published:** 2016-07-22

**Authors:** Hans Michael Hau, Florian Thalmann, Christoph Lübbert, Mehmet Haluk Morgul, Moritz Schmelzle, Georgi Atanasov, Christian Benzing, Undine Lange, Rudolf Ascherl, Roman Ganzer, Dirk Uhlmann, Hans-Michael Tautenhahn, Georg Wiltberger, Michael Bartels

**Affiliations:** Department of Visceral, Transplantation, Vascular and Thoracic Surgery, University Hospital of Leipzig, Liebigstrasse 20, 04103 Leipzig, Germany; Department of General, Visceral and Transplant Surgery, Campus Virchow, Charité-Universitätsmedizin Berlin, Augustenburger Platz 1, Berlin, Germany; Department of Urology, University of Leipzig, Leipzig, Germany; Division of Infectious Diseases and Tropical Medicine, Department of Gastroenterology and Rheumatology, Leipzig University Hospital, Leipzig, Germany

**Keywords:** Liver resection, Metastasic renal cancer, Tyrosinkinase inhibitor agents

## Abstract

**Background:**

The value of liver-directed therapy (LDT) in patients with metastasic renal cell carcinoma (MRCC) is still an active field of research, particularly in the era of tyrosinkinase inhibitor (TKI) therapy.

**Methods:**

The records of 35 patients with MRCC undergoing LDT of metastasic liver lesions between 1992 and 2015 were retrospectively analyzed. Immediate postoperative TKI was given in a subgroup of patients after LDT for metastasic lesions. Uni- and multivariate models were applied to assess overall survival (OS), progression-free survival (PFS) and disease-free survival (DFS).

**Results:**

Following primary tumor (renal cell cancer) resection and LDT, respectively, median OS was better for a total of 16 patients (41 %) receiving immediate postoperative TKI with 151 and 98 months, when compared to patients without TKI therapy with 61 (*p = 0.003*) and 40 months (*p = 0.032*). Immediate postoperative TKI was associated with better median PFS (47 months versus 19 months; *p = 0.023*), whereas in DFS only a trend was observed (51 months versus 19 months; *p = 0.110*).

**Conclusions:**

LDT should be considered as a suitable additive tool in the era of TKI therapy of MRCC to the liver. In this context, postoperative TKI therapy seems to be associated with better OS and PFS, but not DFS.

## Background

Renal cell carcinoma (RCC) is one of the most common tumors with an increasing incidence of approximately 85 new cases and 35 deaths per 100 000 reported in 2012 in Europe [1–3]. The majority of patients with RCC present with an early stage of their disease, 20–30 % have synchronous metastases and 20–50 % of the patients develop metastasic disease after primary tumor resection [4–7].

Most data exist for lung metastases which represent the common sites of distant metastases [5, 8]. Although it is less common, liver metastases were observed in 30–40 % of the patients with metastasic disease [5, 9]. Unfortunately, development of liver metastasis was generally considered as a poor prognostic factor and was frequently associated with more widespread disease [10, 11]. That’s the reason why liver resections in these patients are often single decisions and consequently, there is little available literature on this topic. Although larger series were published with a focus on non-colorectal/non-neuroendocrine metastases, patients with RCC represent only a small subgroup and were not the focus of these studies [10, 12].

Unfortunately, the prognosis is poor in MRCC with 5-year survival ranging from 5 to 15 %, because chemotherapy, radiotherapy or immunotherapy have almost no influence or showed limited response [6, 8, 9, 11, 13–16]. Since several years, antiangiogenic targeted therapies (TT), particularly tyrosinkinse inhibitor (TKI) agents, have become available and showed improved clinical outcomes in large multicenter trials [17–21]. However, a majority of patients treated with TT develop resistance to this drugs and complete response remains extremely rare. Surgical treatment if feasible is the only potentially curative treatment option for patients with MRCC [7, 8, 22]. Complete resection of metastatic lesions is consistently associated with survival benefits as previous studies has noted [23–25]. Thus, some authors reported 25–52 % five-year survival rates in cases of MRCC with complete resection and decreased risk of death from RCC [7, 8, 25–27].

However, little information has been available on the utility and benefit of systemic treatment after complete resection of metastatic lesions. There are only a few reports with small patient numbers reporting low long-term progression-free survival rates after metastasectomy [8, 23, 26, 27]. Most of the studies had a focus on the adjuvant treatment with immunotherapeutic or chemotherapeutic agents [6, 24, 28]. Therefore, understanding the role of surgery in the era of TKI therapy - especially in the neoadjuvant and/or adjuvant multimodal treatment setting - is of increasing importance and interest.

In the present study, we examined besides the efficacy of surgical therapy for MRCC - with a focus to the liver - the different treatment regimens that were received by patients with hepatic metastasized RCC after surgical resections of metastases. In addition, we assessed the effect of immediate postoperative TT on short- and long-term outcome like survival, tumor recurrence and morbidity in patients with MRCC following primary tumor resection and liver-directed therapy, respectively.

## Methods

### Patient selection and study population

Medical records of patients undergoing liver-directed therapy (liver-directed therapy - LDT) for histologically proven RCC liver metastases (RCCLM) at the Department of Surgery, University Hospital of Leipzig, between 1992 and 2015 were analyzed retrospectively. The study was approved by the local ethical commission board from the University of Leipzig (AZ EK: 243-14-14072014). Due to the retrospective design of the study and accordingly national guidelines, the local ethic committee confirmed, that informed consent was not necessary from participants.

Inclusion criteria consist of patients with metastasic RCC to the liver who underwent LDT. Only patients with complete available follow-up data were included in the study. Patients with extended and unresectable liver tumors, peripheral diffuse metastasic disease, or diffuse hepatic involvements of all liver lobes were excluded from study. Resectable synchronous extra-hepatic site metastases were no contraindications for study inclusion provided that the overall surgical strategy aimed to be curative. All patients underwent staging examinations to determine suitability and eligibility for surgery including physical examination, serum laboratory tests, abdominal ultrasound, computed tomography (CT) (abdomen/chest) or magnetic resonance tomography. In some cases the diagnosis of liver metastasis was confirmed by fine-needle cytology or biopsy. The Karnofsky Performance Scale (KPS) of our study population were not less than 80.

All cases of patients with RCCLM were discussed by a multidisciplinary team to assess the indication and suitability for surgery. The decision for use of neoadjuvant and/or immediate adjuvant targeted therapy following liver metastasectomy was made on an individual basis in oncological tumor board and was not prospectively specified. With regards to survival analysis, patients who are withdrawn from a study as well as patients who are lost to follow-up or alive without the event occurrence at latest follow-up were censored in the survival chart.

The primary aim of the present study was to assess the effect of immediate postoperative TT/TKI therapy on progression-free survival (PFS)/disease-free survival (DFS) of patients with hepatic metastasized renal cell cancer who received metastasectomy of primary metastasic lesions or liver-directed therapy of metastatic hepatic lesions, respectively. The secondary endpoint was to assess the overall survival in the different treatment regimes.

### Variables/data collection

Standard clinicopathological and demographic parameters of the study population were collected including: Patient age (years); gender (male versus female); body mass index (kg/m^2^); oncological parameters (Eastern Cooperative Oncology Group (ECOG) performance Status (0–5)); primary tumor characteristics (tumor location (right versus left kidney), T-stage (T1-T4), lymph node stage (N0-N2), Fuhrman grading (low (1–2); high (3–4)), histological subtype (clear cell, chromophobe, papillary/other)); treatment of the primary tumor (type of surgery (complete or partial nephrectomy). Variables were also collected on the number (n), location (unilobular versus multilobular); size (cm), weight (g) of hepatic lesions as well as occurrence of liver metastases (synchronous versus metachronous) as well as presence of extrahepatic metastases (yes versus no). A particular interest applied with details of adjuvant TT/TKI therapy (yes versus no) after occurrence of metastasic lesions and liver resection. Additionally, the different TKI treatment regimes, the onset and duration of TKI therapy following liver-directed therapy as well as the efficacy of TKI therapy were examined.

Operative information including type of liver-directed therapy (surgery, interventional procedure (ablation, electroporation), operating time (minutes), intraoperative transfusion (yes vs. no), red blood cell concentrate (RBCC), fresh frozen plasma (FFP), application of vascular inflow occlusion (Pringles manoeuvre: yes vs. no) and extent of resection. Hepatectomy was classified as minor (less than three segments) and major (three or more segments). Margin status (microscopically complete- R0, microscopic (R1) and macroscopic involvement (R2)) was determined based on final pathological assessment. Peri- and postoperative collected data including length of intensive care unit (ICU) stay (days), length of hospital stay (days), tumor recurrence, survival such as morbidity and mortality rate. Postoperative complications are presented according to the Clavien-Dindo-Classification (grade I to V). Therefore, grades I and II were taken together as minor complications and grade III-V as major complications. The perioperative mortality was defined as 30-day mortality.

Overall survival (OS) was either calculated as the time interval between primary tumor operation (renal cell cancer resection) until latest follow-up/or death or secondary tumor operation (liver tumor therapy) until latest follow-up/or death.

Disease-free interval (DFI) was calculated as the time interval from liver-directed therapy until first recurrence at any site. Progression-free survival (PFS) was calculated from time of resection of first metastases/start of TKI therapy until time of progression/recurrence or last follow-up/death. Disease-free survival (DFS) was calculated from date of liver-directed therapy to first recurrence, distant metastases, last follow-up or death.

### Statistical analysis

All data are presented as either percentage, mean value with range or median value with interquartal range (IQR). Data were analyzed using Student’s *t*-test and Wilcoxon rank sum test for continuous variables, and the chi-squared test or Fisher’s exact test for discrete variables, where appropriate. Disease-free survival, progression-free survival and overall survival were examined using the Kaplan-Meier Method and potential prognostic factors were evaluated in univariate analysis by a two-sided log-rank test. A multivariable Cox proportional hazard regression model was used to identify independent prognostic factors for disease recurrence and survival/mortality. Included tested variables in Cox proportional hazard regression analysis were chosen according to the literature and the primary/secondary aims of the study: immediate postoperative targeted Therapy, ECOG performance status >1, synchronous liver metastases and multiple liver metastases. *P*-values < 0.05 were considered statistically significant. All data were analyzed by using SPSS software (SPSS Inc., Chicago, Illinois, USA, version 19.0).

## Results

### General and oncological characteristics

The outcome of 35 patients (female = 11, male = 24) with a median age of 65 years (IQR: 59–71) at time of LDT was investigated.

The primary tumor location was nearly equally distributed between the patient collective (right kidney; 21 (60 %)/left kidney; 14 (40 %). Each patient underwent curative surgery for the primary tumor: radical nephrectomy in 31 patients (86 %) and partial nephrectomy in 4 patients (14 %). The majority of patients (60 %) had lymph node metastases associated with the primary tumor (N1; 40 %, N2; 20 %) and most primary tumors were pathologic T2 or T3 lesions (T1: 20 %; T2: 25.8 %; T3, 42.8 %; T4, 11.4 %). The primary tumor could be removed completely in all patients. Fuhrman Grading was low in most patients (grade 1: 2 patients (5.7 %); grade 2: 22 patients (62.9 %); grade 3: 9 patients (25.7 %); grade 4: 2 patients (5.7 %)). The final histology of the primary tumor showed in all patients RCC, compromising a clear cell subtype in 25 patients (71.4 %), followed by chromophobe carcinoma in 6 (17.1 %) and unclassified/papillary subtype in 3 cases (11.5 %). Prognostic criteria using ECOG performance status were 0 in 29 patients (83 %) and >1 in the remaining 6 patients (17 %). The complete list of patient characteristics is shown in Table 1 without relevant differences regarding general and oncological characteristics between the groups.

**Table 1:** Basic general and oncological characteristics according to state of postoperative Tyrosinkinase Inhibitor (TKI) therapy

Variables	Overall (*n* = 35)	TKI (+) (*n* = 16)	TKI (-) (*n* = 19)	*p*-value
Median Age (years)	65 (59–71)	64.5 (58–71)	69 (62–71)	0.160^a^
Gender	24:11	12:4	12:7	0.452^d^
Male/female	(68.6/31.4 %)	(34.3:11.4 %)	(34.3:20 %)	
Median Body mass index (kg/m2)	26.5 (24.5–28.0)	26.9 (26.0–28.3)	25.9 (24.2–27.7)	0.349^a^
ECOG performance status (%)				
0	29 (82.9 %)	15 (42.9 %)	14 (40 %)	0.117^d^
1	6 (17.1 %)	1 (2.9 %)	5 (14.3 %)	
Primary tumor characteristics				
Right side/left side; n (%)	21/14 (60 %:40 %)	7/9 (20 %:25.7 %)	14/5 (40 %:14.3)	0.072^d^
Primary tumor histology				
Clear cell	25 (71.4 %)	10 (28.6 %)	15 (42.9 %)	0.283^c^
Non-clear cell	10 (28.6 %)	6 (17.1 %)	4 (11.5 %)	
Primary tumor T-stage				
Stage 1–2	16 (45.8 %)	8 (22.9 %)	8 (22.9 %)	0.640^c^
Stage 3–4	19 (54.2 %)	11 (31.3 %)	8 (22.9 %)	
Primary tumor grading				
Low (Fuhrmann 1–2)	24 (68.6 %)	12 (34.3 %)	12 (34.3 %)	0.452^d^
High (Fuhrmann 3–4)	11 (31.4 %)	4 (11.4 %)	7 (20 %)	
Liver tumor distribution				
Unilobular/Multilobular	28:7 (80 %:20 %)	14:2 (40 %:14.3 %)	14:5 (40 %:5.7 %)	0.309^d^
Median tumor size (cm)	4.4 (2.9–6.0)	3.9 (2.5–4.5)	5.6 (3.0–7.5)	0.094^a^
Mean number of hepatic tumor lesions	1.70 (1–5)	1.64 (1–4)	1.74 (1–5)	0.813^b^
Median weight of resected liver (g)	212.5 (39.2–652.5)	81.4 (15.5–265.0)	290 (70–916.3)	0.138^a^
Timing of liver metastases				
Synchronous/	6 (17.1 %)	4 (11.4 %)	2 (5.7 %)	0.258^d^
metachronous	29 (82.9 %)	12 (34.3)	17 (48.6 %)	
Extrahepatic disease at time of liver-directed therapy				
Yes	7 (20 %)	5 (14.3 %)	2 (5.7 %)	0.127^d^
No	28 (80 %)	11 (31.4 %)	17 (48.6 %)	

Used statistical tests:

^a^Student’s *t*-test

^b^Wilcoxon rank sum test

^c^chi-squared test

^d^Fisher’s exact test

Most RCCLM were metachronous (*n* = 29; 82.9 %); the median time interval between the diagnosis of primary tumor and the occurrence of first metastases was 13 months (IQR: 3–36 months), 15 months (IQR: 3–39 months) between primary tumor operation and occurrence of liver metastases. The median time between last staging and liver surgery was 1 month (IQR: 0–2 months). The mean number of hepatic metastatic lesions was 1.7 (range: 1–5) being localized unilobular in the liver (*n* = 28, 80 %). The median size of the largest lesion was 4.4 cm (IQR: 2.9 – 6.0 cm).

### Details of liver-directed therapy

Among the 35 patients with RCCLM, liver-directed treatment consisted of liver resection in 33 patients (94.3 %) and local ablation in 2 patients (5.7 %) (Table 2). In the resection-group, 16 of 35 patients (48.5 %) underwent major liver resections, in 17 patients (51.5 %) minor liver resections were performed. On final pathologic analysis, local R0 (margin free) resection could be achieved in 30 patients (85.7 %); only 5 patients (14.3 %) had microscopic disease at the margin (R1) and none patient had macroscopic disease (R2).

**Table 2:** Operative and perioperative details at time of liver-directed therapy of our study population (*n* = 35 patients) according to state of Tyrosinkinase Inhibitor (TKI) Therapy

Variables	Total (*n* = 35)	TKI+ (*n* = 16)	TKI- (*n* = 19)	*p*-value
Extent of resection				
Minor resection	17 (51.5 %)	11 (33.3 %)	6 (18.2 %)	0.008^d^
Major	16 (48.5 %)	3 (9.1 %)	13 (39.4 %)	
Type of liver resection				
Segmental resection	9 (27.3 %)	6 (18.2 %)	3 (9.1 %)	0.13^d^
Anatomical right/left resection	11 (33.3 %)	9 (27.2 %)	2 (6.1 %)	0.003^d^
Extended resection right/left	5 (15.2 %)	4 (12.1 %)	1 (3.1 %)	0.06^d^
Atypical resection	8 (24.2 %)	4 (12.1 %)	4 (12.1 %)	0.61^d^
Resection Margin				
Complete Resection (R0)	30 (85.7 %)	14 (40 %)	16 (45.7 %)	0.78^d^
Microscopically positive margin (R1)	5 (14.3 %)	2 (5.7 %)	3 (8.6 %)	
Transfusion intraoperative				
Substitution	15 (8 %)	9 (4.8 %)	3 (1.6 %)	0.7^d^
- Transfusion erythrocyte concentrations	0.27 (0–6)	0.23 (0–6)	0.36 (0–4)	0.7^d^
- Transfusion Fresh frozen plasma	0.17 (0–8)	0.2 (0–8)	0.03 (0–1)	0.7^d^
Pringle manoeuvre	19 (54.3 %)	6 (17.1 %)	13 (37.1 %)	0.067^c^
Median Hospital stay (days)	18.7 (11–28)	16.6 (11–18)	20.5 (16–24)	0.360^a^
Median Intensive care unit stay (days)	1.9 (1–6)	2.0 (1–6)	1.8 (1–5)	0.716^a^
Median Operating time (min)	238 (161.5–276)	224.5 (164.5–272.5)	242 (155–281)	0.477^a^
Complications				
Grade I	0 (0 %)	0 (0 %)	0 (0 %)	-
Grade II	3 (8.6 %)	2 (5.7 %)	1 (2.9 %)	0.434^d^
Grade IIIa	5 (14.3 %)	4 (11.4 %)	1 (2.9 %)	0.096^d^
Grade IIIb	1 (2.9 %)	0 (0 %)	1 (2.9 %)	0.352^d^
Grade Iva	1 (2.9 %)	1 (2.9 %)	0 (0 %)	0.269^d^
Grade IVb	0 (0 %)	0 (0 %)	0 (0 %)	-
Grade V	0 (0.5 %)	0 (0 %)	0 (0 %)	-

Used Statistical tests:

^a^Student’s *t*-test

^b^Wilcoxon rank sum test

^c^chi-squared test

^d^Fisher’s exact test

Median operating time was 238 min (IQR: 161.5–276 min), median ICU and hospital stay were 1.9 days (IQR: 1–6 days) and 18.7 (IQR: 11–28 days), respectively. Postoperative complications were classified according to the Clavien-Dindo Classification, with an overall postoperative morbidity in 10 patients (28.5 %). In this context, minor complications (grade I, *n* =0; 0 %; grade II, *n* = 3; 8.6 %) were observed in 3 patients (8.6 %), major complications occurred in 7 patients (19.9 %) (grade IIIa, *n* = 5, 14.3 %; grade IIIb, *n* = 1, 2.9 %; grade IV, *n* = 1, 2.9 %; grade V, *n* = 0; 0 %). The 30-day mortality of the study group was 0 %.

### Treatment after resection of metastatic lesions/recurrence analysis

At time of liver-directed therapy, the liver was the first and only metastases in 28 patients (*n* = 80 %). Whereas, there was evidence of extrahepatic disease in 7 patients (20 %). These metastases were surgically treated with curative intention before liver resection, or were synchronously discovered at laparotomy and subsequently resected. The most common sites of metastases were the retroperitoneum/local recurrence (*n* = 5; 14.3 %), the lung (*n* = 2; 5.7 %), the adrenal glands (*n* = 2; 5.7 %) and the bone (*n* = 1; 2.8 %).

**Fig. 1:**
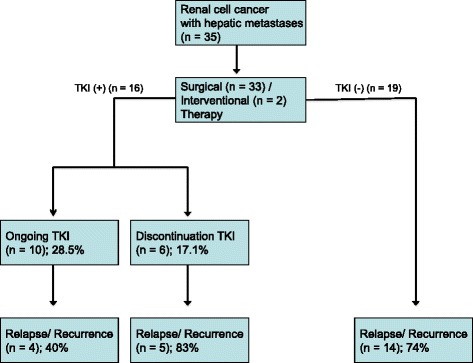
Treatment algorithm of patients with hepatic metastasized renal cell cancer following liver-directed therapy

A total of 16 patients (41 %) received TKI therapy following liver-directed therapy of their metastatic hepatic lesions after a median duration of 2.4 months (IQR: 1–5 months) (Fig. 1). In 3 patients a preoperative targeted therapy was started with median duration of 2.1 months (IQR: 1–25 months) and continued after liver-directed therapy. The given targeted therapy included sunitinib (*n* = 10 patients; 62.5 %), sorafenib (*n* = 2 patients; 12.5 %), pazopanib (*n* = 3 patients; 18.8 %) and everolimus (*n* = 1 patient; 6.2 %).

Of these 16 patients, 10 patients (28.5 %) had ongoing TT after liver-directed therapy for a median duration of 21 months (IQR: 18–49 months) and 6 patients (17.1 %) discontinued TT therapy at a median duration of 13 months (IQR: 7–35 months) without evidence of recurrence. Of the 10 patients with ongoing TT after liver-directed therapy, 4 patients (40 %) experienced relapse/recurrence. Of the 6 patients discontinued TT, 5 patients (83 %) experienced relapse/recurrence.

Of the 19 patients (59 %), who did not receive immediate postoperative targeted therapy, 14 patients (74 %) experienced recurrence after a median time of 11 months (IQR: 4–31 months). In total, 23 patients (66 %) did develop recurrence following LDT within a median time of 16 months (IQR: 5–34 months). Among these patients, the pattern of recurrence was extrahepatic in 13 patients (57 %), intrahepatic in 7 patients (30 %) and both in 3 patients (13 %). At the end of the observation period, 20 patients (57 %) had died. Of the 15 patients (43 %) alive, 8 patients (54 %) remained free of recurrence after a median follow-up of 2.1 years (range: 1–4.3 years). Of the 7 patients (43 %) developed recurrence, only 2 patients had local metastases in the pre-existing site. Lung metastases were developed most frequently (*n* = 4), followed by the liver (*n* = 2) and the bone (*n* = 1). Sites of recurrence were not different according to the immediate postoperative TKI therapy.

Median progression-free survival and median disease-free survival was 27 months (IQR: 8–47 months; median follow-up was 3.1 years (IQR: 1.1–4.8 years)) following resection of first metastatic lesions and 19 months (IQR: 7–58 months; median follow-up was 2.1 years (IQR: 1–4.3 years)) following LDT, respectively. One-, 3-, and 5-year progression-free survival following resection of first metastases was 60, 28.6 and 11.4 %, respectively. Following LDT, 1-, 3- and 5-year disease-free survival was 60, 22.9 and 14.3 %, respectively.

**Fig. 2:**
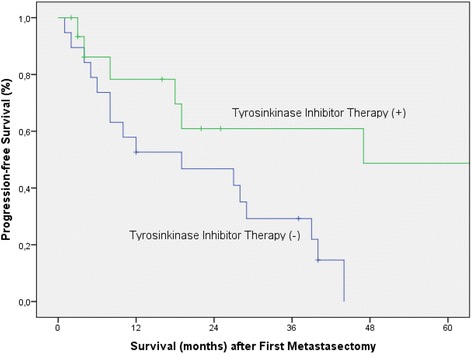
Kaplan-Meier survival curve representing progression-free survival after first metastasectomy according to immediate postoperative tyrosinkinase inhibitor therapy (*p* = 0.023)

In univariate analysis for progression-free survival, immediate postoperative targeted therapy (*p = 0.023*) (Fig. 2), ECOG performance status 0 (*p = 0.015*), metachronous liver metastases (*p = 0.035*), negative resection margins (*p = 0.001*), T-stage 1/2 (*p = 0.027*) and low Fuhrman Grading (*p = 0.002*) were associated with significantely longer progression-free survival rates (Table 3). At multivariable Cox regression analysis, immediate postoperative targeted therapy (HR 0.32, 95 CI: 0.1–0.9, *p = 0.032*) could be identified as a favourable significant independent predictor of good progression-free survival. In contrast, ECOG performance status >1 (HR 3.4, 95 CI 1.2–9.8, *p = 0.023*) could be identified as independent significant predictor associated with disease progression.

**Fig. 3:**
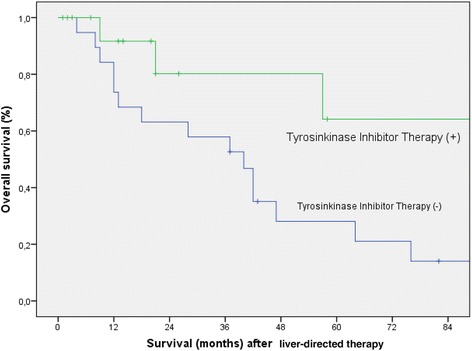
Kaplan-Meier overall survival curve representing follow-up after liver-directed therapy according to immediate postoperative tyrosinkinase inhibitor therapy (*p* = 0.032)

**Table 3:** Univariate analysis of potential prognostic factors for progression-free survival (*n* = 35 patients) and disease-free survival (*n* = 35 patients) following metastasectomy of primary and hepatic metastasic lesions, respectively

		Progression-free Survival after first Metastasectomy		Disease-free Survival after Liver-directed therapy (LDT)	
Variables	n	Median Time (months) with IQR	*p-value*	Median Time (months) with IQR	*p-value*
Gender					
Male	24	8 (5–44)	0.324	15 (5–44)	0.346
Female	11	27 (10–47)		20 (9–58)	
Targeted therapy					
TKI+	16	47 (18–79)	**0.023**	58 (18–79)	0.110
TKI–	19	19 (6–39)		19 (4–46)	
ECOG					
0	29	29 (10–47)	**0.015**	20 (8–71)	0.132
> 1	6	6 (2–28)		15 (1–28)	
Age					
< 65 years	18	19 (8–40)	0.518	19 (5–71)	0.960
> 65 years	17	39 (8–71)		20 (9–58)	
Tumor distribution					
unilobular	28	28 (8–44)	0.772	28 (8–58)	0.981
multilobular	7	19 (3–77)		19 (3–79)	
Extrahepatic Disease					
Yes	28	27 (7–45)	0.412	19 (8–59)	0.962
No	7	29 (4–82)		20 (4–29)	
Liver metastasis Timing					
Synchronous	6	4 (2–9)	**0.035**	4 (2–8)	0.085
Metachronous	29	28 (10–47)		26 (12–58)	
Tumor size, cm					
< 4.5 cm	21	39 (10–71)	0.07	19 (9–42)	0.376
> 4.5 cm	14	18 (6–29)		18 (5–35)	
Resection margin					
Radical resection (R0)	30	28 (12–51)	**0.001**	28 (12–58)	**0.014**
Irradical resection (R1/R2)	5	6 (3–10)		4 (3–9)	
Number of metastases					
Solitary	22	27 (8–47)	0.653	28 (9–51)	0.967
Multiple	13	19 (8–40)		19 (5–79)	
Grading of RCC					
Low (G1/G2)	24	40 (18–71)	**0.002**	44 (18–71)	**0.001**
High (G3/G4)	11	8 (5–19)		8 (4–19)	
T-stage					
T1/T2	16	44 (12–79)	**0.027**	29 (15–71)	0.071
T3/T4	19	18 (6–39)		18 (4–51)	
Disease-free intervall					
< 12 months	17	28 (5–79)	0.753	28 (5–79)	0.501
> 12 months	18	27 (10–40)		19 (9–58)	

*TKI* Tyrosinkinase InhibitorTherapy, *ECOG* Eastern Cooperative Oncology Group

The bold data specifies a staticial significance *p*-values < 0.05

Following factors show benefitial effects on disease-free survival in univariate analyses: Negative resection margins (*p = 0.014*) and low Fuhrman grading (*p = 0.001*) (Table 4). Immediate postoperative TKI therapy (*p = 0.1*), ECOG status 0 (*p = 0.1*), metachronous liver metastases (*p* = 0.08) and T-stage 1/2 (*p = 0.07*), however, showed only a trend for a decreased recurrence rate in univariate analysis.

**Table 4:** Univariate analysis of potential prognostic factors for overall Survival (*n* = 35 patients) following primary tumor resection and liver directed therapy (LDT) , respectively

		Follow-Up Liver-directed therapy		Follow-Up Primary Tumor Resection	
Variables	n	Median Time (months) with IQR	*p-value*	Median Time (months) with IQR	*p-value*
Gender					
Male	24	42 (21–98)	0.7	96 (34–130)	0.4
Female	11	37 (12–68)		62 (63–126)	
Targeted therapy					
TKI+	16	98 (57–133)	**0.032**	151 (126–202)	**0.003**
TKI−	19	40 (12–64)		61 (25–103)	
ECOG					
0	29	57 (37–98)	**0.001**	103 (79–151)	**<0.001**
> 1	6	12 (9–18)		15 (13–34)	
Age					
< 65 years	18	64 (35–98)	0.07	96 (34–151)	0.31
> 65 years	17	40 (12–57)		63 (79–126)	
Tumor distribution					
unilobular	28	98 (21–76)	0.911	126 (25–151)	0.8
multilobular	7	42 (12–98)		86 (54–126)	
Synchronous Extrahepatic Disease					
Yes	7	21 (9–37)	0.312	63 (54–126)	0.727
No	28	47 (28–98)		86 (61–159)	
Liver metastasis Timing					
Synchronous	6	9 (8–12)	**0.006**	15 (8–63)	**0.006**
Metachronous	29	47 (28–98)		96 (54–151)	
Tumor size, cm					
< 45 mm	21	98 (28–133)	**0.007**	103 (86–151)	**0.007**
> 45 mm	14	37 (12–57)		63 (18–85)	
Resection margin					
Radical resection (R0)	30	47 (21–98)	**0.013**	96 (48–151)	**0.041**
Irradical resection (R1/R2)	5	9 (8–28)		86 (13–92)	
Number of metastases					
Solitary	22	64 (12–98)	0.850	85 (54–153)	0.990
Multiple	13	42 (21–76)		116 (34–130)	
T-stage					
T1/T2	16	47 (37–98)	0.062	126 (62–151)	0.257
T3/T4	19	40 (12–64)		85 (54–103)	
Grading of RCC					
Low (G1/G2)	24	47 (37–133)	0.543	116 (63–126)	0.501
High (G3/G4)	11	28 (9–98)		85 (51–151)	
Disease-free intervall					
< 12 months	17	13 (9–42)	**0.002**	79 (18–96)	0.2
> 12 months	18	64 (42–98)		103 (62–151)	
Histology					
Clear cell	25	47 (40–98)	0.821	96 (51–151)	0.498
Non clear cell	10	42 (18–132)		85 (54–116)	
Surgical complications					
Yes	10	28 (21–40)	0.996	85 (49–118)	0.104
No	25	47 (13–98)		151 (54–202)	

*TKI* Tyrosinkinase InhibitorTherapy, *ECOG* Eastern Cooperative Oncology Group

The bold data specifies a staticial significance *p*-values < 0.05

### Survival analysis

Median overall survival was 86 months (IQR: 21 – 136 months; median follow-up was 5.1 years (IQR: 1.8 – 8.0 years) following primary tumor resection and 42 months (IQR: 18 – 98 months; median follow-up was 2.2 years (IQR: 1 – 4.8 years) following LDT, respectively. One-, 3-, and 5-year survival following resection of the primary tumor was 88.6, 68.6 and 51.4 %, respectively. Following LDT, 1-, 3- and 5-year survival was 77.1, 45.7 and 20 %, respectively.

In the univariate analysis using the Kaplan-Meier Method, immediate postoperative TKI therapy (*p* = *0.032*) (Fig. 3), ECOG performance status 0 (*p < 0.001*), metachronous liver metastases (*p = 0.006*), liver tumor size <45 mm (*p = 0.007*), negative resection margins (*p = 0.013*) and a disease free survival >12 months (*p = 0.002*) were significant predictors of good overall survival following liver-directed therapy (Table 4).

Multivariate survival analysis using a Cox regression model showed that immediate postoperative TKI therapy (HR 0.28, 95CI: 0.1–0.99, *p* = *0.046*) was a favourable significant independent predictor of good overall survival. Whereas ECOG performance status >1 (HR 4.6, 95 CI 1.4–14.7, *p < 0.001*) and synchronous liver metastases (HR 5.7 95 CI 1.4–23.8, *p* = *0.015*) are significant independent predictors of poor overall survival. Whereas, multiple liver metastases (HR 1.1; 95 CI 0.4–2.9, *p* = 0.8) were no significant independent predictors of overall survival.

Following primary tumor resection, in univariate analysis immediate postoperative TKI therapy (*p = 0.003*), ECOG performance status 0 (*p < 0.001*), metachronous liver metastases (*p = 0.006*), liver tumor size <45 mm (*p = 0.007*) and negative resection margins (*p = 0.041*) were of prognostic impact on good overall survival. Multivariate analysis using a Cox regression model confirmed immediate postoperative TKI therapy (HR 0.14, 95 CI: 0.03–0.6, *p* = *0.011*) as a favourable significant independent predictor of good overall survival. In contrast, ECOG performance status >1 (HR 8.3, 95 CI 1.9–18.7, *p < 0.001*) and synchronous liver metastases (HR 5.4 95 CI 1.1–26.8, *p* = *0.013*) are significant independent predictors of poor overall survival.

## Discussion

To our best knowledge, this is one of the largest studies examining the value of metastasectomy with a special focus on liver resection in patients with MRCC in the era of TKI therapy. We examined whether TKI therapy is associated with MRCC prognosis when it is provided immediately after surgical resection of metastatic hepatic lesions. Uni- and multivariate analyses showed that median overall survival such as progression-free survival were significantly better in patients receiving immediate postoperative TKI therapy following primary tumor resection and LDT, respectively. However, disease recurrence following liver resection showed only a trend to be less common in patients who received immediate postoperative TKI therapy. These results could be a sign that immediate postoperative TKI therapy improves survival and disease control by decreasing tumor recurrence following resection of metastatic hepatic lesions.

Various treatment options have been studied for the therapy of MRCC and the benefits of metastasectomy in these patients could be demonstrated in some retrospective studies [7, 8, 29]. In this context, there is emerging evidence that surgical resection may represent a significant tool in multimodal concepts of MRCC. In accordance with retrospective studies, our study demonstrates liver resection to be safely applicable with comparable survival and recurrence rates for patients with resectable RCCLM [6, 10, 11, 13, 30]. Therefore, selected clinical characteristics and patient-defined variables have been determined to stratify risk for metastasectomy in MRCC patients to help to define groups of patients which benefit most from surgical therapy with a focus on liver metastasectomy. In accordance with previous reports, in our study immediate postoperative TKI therapy, ECOG performance status, timing of liver metastases presence, tumor diameter, primary tumor characteristics (Fuhrman Grading, T-Stage,), negative resection margins and time of disease free interval were of prognostic value for overall survival and recurrence [6, 10, 11, 25, 30–33].

However, the prognostic value of Fuhrman grading in papillary renal cancer is inconsistent and nowdays not considered as the best grading system for non-clear cell renal cancer. In recent studies, nuclear grade seems to be more effective to predict outcome in patients with non-clear cell renal cancer [34]. Other factors like patients’ age, number of metastases, presence of synchronous extrahepatic disease such as factors related to tumor biology might help to decide to operate or not.

Overall, approximately 70 % (25/35) of our patients experienced disease recurrence after surgical resection of first metastatic lesions. Our results are consistent with previous studies which have shown that large numbers of patients experience disease recurrence after metastasectomy with excellent cancer-specific overall survival. Johansen et al. reported that disease recurrence was observed in 66.7 % of patients, with complete response achieved by targeted therapy alone or no evidence of disease with additional metastasectomy after a median follow up of 12 months [35]. More recently, Park et al. reported about disease recurrence in 60.4 % of patients received immediate postoperative targeted therapy after metastasectomy or not over a median follow-up of 12 months [27]. These results confirmed the fact that some residual cancer cells might remain after the complete resection of metastatic lesions.

Some clinical trials showed that advanced RCC is often resistant to most systemic therapies, and shows limited response to immunotherapy [8, 16]. Recently, several randomized controlled trials have shown that TKI like sorafenib, sunitinib or pazopinib, and other targeted therapeutical agents are correlated with improvements in PFS and OS [20, 21, 36, 37]. These new drugs have dramatically changed the treatment and prognosis of patients with MRCC, and give the opportunity to perform metastatic resection in a new and expended population of patients as more response has been noted. However, cure is rare with TKI alone and complete remission after TKI treatment occurs in only 1–3 % of cases [18, 19]. Whereas, partial response could be achieved in 10–39 % of patients, and ceiling of the overall survival was 18.8 months (range 7.8 to 43.2 depending of the risk factors) [35, 38, 39]. Therefore, metastasectomy remains an important treatment for MRCC and the growing evidence of long-term response with TKI therapy has been stimulating multimodal approaches in the last years.

However, there is currently low experience with the role of systemic TT after surgical treatment of metastatic lesions in patients with RCC and experience is based on case reports and small series [8, 22, 26, 40]. Reliable data examining patients with RCCLM following liver resection are completely missing. The largest series was published recently by Park et al. who examined the efficacy of adjuvant targeted therapy after complete resection of metastatic lesions in 53 patients with MRCC; however none of the patients had RCCLM. 19 (35.9 %) of the patients received immediate postoperative targeted therapy. Of the 34 who did not receive immediate postoperative targeted therapy, 27 (79.4 %) experienced disease recurrence. Immediate postoperative targeted therapy was associated with better median progression free survival (not reached versus 20.0 months, *p = 0.017*), but not cancer-specific survival [27]. According to the missing available data, prospective, randomized multicentre clinical trials (RESORT Study and SMAT-AN 20/04 of the Working Group of Urological Oncology (AUO)) were initiated within the last years to studying the effects and benefits of targeted adjuvant therapy following metastasectomy [18, 41, 42].

Nevertheless, there are some limitations to our study. Firstly, it concerns a small patient sample and due to the retrospective nature of our study it is susceptible to bias in data selection and survival analysis. Although our results show significant differences in overall survival (OS) and progression-free survival (PFS), we could not find significant differences in disease-free survival (DFS), which at first sight might seem astonishing. The three outcome variables are very distinct from each other and may not readily be compared as they include different patients. Also, regarding the trends in the data, the follow up might have been to short to show differences in terms of DFS. Unfortunately, the sample size was too small to allow a final answer to our hypotheses.

Secondly, there are no comparative studies in this highly selected patient population which examine patients with MRCC without surgical intervention who were treated with targeted therapy. Thirdly, there were no standard protocols for the clinical decision making process concerning the choice of given targeted agents as well as the timing of restarting or discontinuating the targeted therapy. This fact is especially mirrored in our study because of the long observation period of 23 years. For our first patients TKI therapy was not yet available. During the next period, several publications about the use of TKI in different clinical settings influenced the decision of our interdisciplinary tumor board to use or avoid additional TKI after surgery, respectively. Furthermore, in patients with R1 resection and higher tumor stages the decisions in favour of using TKI were influenced more positively compared to lower tumor stages. Therefore differences in survival might also be due to some selection bias. Of interest, data on patient Quality of Life (QoL) might be an important addition in future studies for the results of our analysis.

## Conclusions

The present data suggest that a surgical approach of MRCC to the liver in combination with adjuvant TKI therapy should be carefully considered case-by-case, taking into account patient QoL in order to optimize the management of these patients. However, future prospective studies, preferably with randomized design and larger populations, are needed do increase the quality of evidence regarding the role of (liver-)metastasectomy with integration of TKI therapy in the (neo)/-adjuvant setting. Moreover, a careful balance of the benefit/risk ratio must be shared among a multidisciplinary team to tailor treatments individually and with the patient. Therefore, prognostic models and algorithms might indeed help in deciding which patients benefit from (liver-) metastasectomy as an additive tool in the multimodal therapeutical setting of MRCC.

## Abbreviations

CT, computed tomography; DFI, disease-free interval; DFS, disease-free survival; ECOG, eastern cooperative oncology group; FFP, fresh frozen plasma; HR, hazard ratio; ICU, intensive care unit; IQR, interquartal range; KPS, karnofsky performance scale; LDT, liver-directed therapy; MRCC, metastasic renal cell carcinoma; OS, Overall Survival; PFS, progression-free survival; QoL, quality of life; RBCC, red blood cell concentrate; RCC, renal cell carcinoma; RCCLM, renal cell carcinoma liver metastases; TKI, tyrosinkinase inhibitor; TT, Targeted Therapy
